# Postbiotics in the medical field under the perspective of the ISAPP definition: scientific, regulatory, and marketing considerations

**DOI:** 10.3389/fphar.2023.1239745

**Published:** 2023-09-08

**Authors:** Gabriel Vinderola, Céline Druart, Luis Gosálbez, Seppo Salminen, Nina Vinot, Sarah Lebeer

**Affiliations:** ^1^ Instituto de Lactología Industrial (CONICET-UNL), Faculty of Chemical Engineering, National University of Litoral, Santa Fe, Argentina; ^2^ Pharmabiotic Research Institute, Narbonne, France; ^3^ Sandwalk Bioventures, Madrid, Spain; ^4^ Functional Foods Forum, Faculty of Medicine, University of Turku, Turku, Finland; ^5^ TargEDys, Longjumeau, France; ^6^ Department of Bioscience Engineering, Research Group Environmental Ecology and Applied Microbiology, University of Antwerp, Antwerp, Belgium

**Keywords:** postbiotics, International Scientific Association for Probiotics and Prebiotics, ISAPP, medicinal product, medical device, drug

## Abstract

Diverse terms have been used in the literature to refer to the health benefits obtained from the administration of non-viable microorganisms or their cell fragments and metabolites. In an effort to provide continuity to this emerging field, the International Scientific Association of Probiotics and Prebiotics (ISAPP) convened a panel of experts to consider this category of substances and adopted the term postbiotic, which they defined as a “preparation of inanimate microorganisms and/or their components that confers a health benefit on the host.” This definition does not stipulate any specific health benefit, finished product, target population or regulatory status. In this perspective article, we focused on postbiotics developed for pharmaceutical uses, including medicinal products and medical devices. We address how this field is regulated for products based on inanimate microorganisms, marketing considerations and existing examples of postbiotics products developed as cosmetics for the skin, for vaginal health, and as orally consumed products. We focus on the European Union for regulatory aspects, but also give examples from other geographical areas.

## 1 Introduction

The beneficial effects of non-viable microorganisms and their fermentation products or metabolites have been documented over the past decades, as evidenced, for example, by a review performed 25 years ago comparing the health benefits of fermented milks carrying viable or non-viable bacteria (Ouwehand and Salminen, 1998). However, over the history of published papers on this topic, a diversity of terms has been used to refer to non-viable microorganisms or their cell fragments, such as inactivated probiotics, heat-inactivated probiotics, non-viable probiotics, dead probiotics, tyndallized probiotics, ghost probiotics, paraprobiotics, postbiotics, cell fragments, and cell lysates (Vinderola et al., 2022a). The lack of a uniform terminology presents several drawbacks for the field, including the challenge of how to search existing evidence to perform systematic reviews and meta-analysis, the communication to stakeholders (consumers, health practitioners, industry) and, perhaps most importantly, coherent and harmonized regulatory frameworks. To coalesce all these terms into a single term and propose a consensus definition and a scope, the International Scientific Association of Probiotics and Prebiotics (ISAPP) adopted the term postbiotic and defined it as “preparation of inanimate microorganisms and/or their components that confers a health benefit on the host” (Salminen et al., 2021a). A strong rationale for the choice of the term postbiotic over the other terms has been articulated (Salminen et al., 2021a; 2021b). In brief, postbiotic is a term composed of ‘post,’ meaning after, and ‘bios,’ meaning life. Therefore, the term postbiotic appropriately refers to a microorganism that has been treated so that it lacks of metabolic capacity and/or procreative potential in conditions conducive to that microorganism’s metabolism and growth. Further, the terms that required that a postbiotic be developed from a probiotic were not preferred as this imposes an unnecessary burden to product development: a microbe must be studied first for its probiotic properties before conducting the necessary studies to demonstrate it is effective on its non-viable form. The terms specifying only cell fragments or cell lysates do not include whole intact non-viable microbes.

The term preparation was included in the definition to anticipate the fact that the viability termination technology used (for example, heat, high pressure, or radiation) may have an impact on functionality, as was demonstrated when different life termination technologies were compared (Wong and Ustunol, 2016), and to anticipate that metabolites and cell fragments could be present, or not (in case microbes are filtered to eliminate metabolites and growth medium components). Then, the term preparation makes the definition wide enough to reflect present developments discussed below and to accommodate innovation. So far, the concept of postbiotics has been applied mainly to food and food supplements intended for healthy populations (Vinderola et al., 2022). However, as is the case for all ISAPP-defined biotic substances [probiotics (Hill et al., 2014), prebiotics (Gibson et al., 2017), synbiotics (Swanson et al., 2020) and postbiotics (Salminen et al., 2021a)] the definition is not proscriptive with regard to specific health benefit, target population (healthy individuals/patients with a specific disease), finished product type, or specific regulatory status. Based on these parameters, the definitions proposed by ISAPP cover “substances” that are found in a large range of finished products, from food to medicinal products or medical devices (Table 1). The aim of this perspective article is to illustrate the main regulatory differences for a non-viable microorganism, able to confer a health benefit, targeted for food or pharmaceutical uses, and to illustrate present and potential applications of postbiotics as food supplements, cosmetic products, medicinal products or medical devices.

**TABLE 1:** Comparison of European regulatory statuses applicable to finished products containing postbiotics: food ingredients, food supplements, medicinal products and medical devices.

	Food ingredients and supplements	Medicinal products	Medical devices
Targeted population	Healthy, general population	Patients suffering from a particular disease	Patients suffering from a particular disease
Intended use	Meet the nutritional needs of the general population	Prevent or treat a disease, alleviate symptoms, make a medical diagnosis	Diagnose, prevent, monitor, predict, prognose, treat, or alleviate disease
Mechanism of action	Nutritional or physiological	Pharmacological, immunological, or metabolic	Non- pharmacological, immunological, or metabolic means (i.e., physical)
Claims	Health claims	Disease claims	Disease claims
Requirements to reach the market	Historical safe consumption or demonstration of safety in the case of “novel food”	Demonstration of safety, quality, and efficacy à positive benefit/risk ratio in the targeted population	Demonstration of safety, quality, and efficacy à positive benefit/risk ratio in the targeted population
Production/quality requirements	HACCP; ISO 22000; FSSC 22000	pharma GMP	CE marking
European competent authority	EFSA	EMA	Notified bodies

## 2 Examples of next-generation postbiotics

The ISAPP definition does not require that the progenitor strain of a postbiotic be a probiotic. A microbe of interest could potentially be developed and marketed as an inanimate microorganism first, whether or not its live counterpart is approved for human consumption. This is the case of pasteurised *Akkermansia muciniphila*. *A. muciniphila* was first isolated as a highly abundant mucus degrader in healthy adults and later described in all age groups in varying amounts. This species is especially low in obese people or in people displaying metabolic syndrome (Cani et al., 2022). *Akkermansia* was both a novel genus and new species and therefore it underwent safety evaluation in the live form for the Qualitative Presumption of Safety (QPS) status in the European Union. Viable *Akkermansia* was evaluated and not recommended for the QPS status due to safety concerns (https://www.efsa.europa.eu/en/efsajournal/pub/5965). In addition, even before the evaluation for inclusion of the live form of *A. muciniphila* in the QPS list, the pasteurised form was already submitted for Novel Food safety evaluation in the EU. Safety evaluation was undertaken as required for novel food regulation as interest in inanimate form had indicated beneficial properties in human studies (Depommier et al., 2019). Novel Food authorisation was received in 2021. This was necessary because there was no “significant” consumption by humans in the European Union before May 1997. This pasteurised form of *A. muciniphila* fits the concept of a postbiotic. *Akkermansia muciniphila* has been reported to have anti-inflammatory properties both in the postbiotic (inanimate) and live form (Cani et al., 2022). Pasteurised *A. muciniphila* is intended to be used in food supplements as defined in EU Directive 2002/46/EC, and in foods for special medical purposes as defined in Regulation (EU) No 609/2013. The applicant requested data protection according to the provisions of Article 26 of Regulation (EU) 2015/2283, and this was granted. Data protection means that newly developed scientific evidence or scientific data supporting the application shall not be used for the benefit of a subsequent application during a period of 5 years from the date of the authorisation of the novel food without the agreement of the initial applicant. In other words, data supporting the novel food application cannot be used in another novel food dossier during a period of 5 years. However, developers are free to gather their own data and submit a novel food dossier to put a product on the market. Indeed, data protection does not mean market exclusivity.

Interest was focused on *Faecalibacterium prausnitzii* too, as another abundant bacterial species found in the healthy human gut. Additionally, it has been suggested to have a potentially important role in promoting gut health (Martín et al., 2017). *F. prausnitzii* was the only species in the genus since its identification. However, two novel *Faecalibacterium* species, i.e., *Faecalibacterium butyricigenerans* sp. nov. and *Faecalibacterium longum* sp. nov., were described later (Zou et al., 2021). These two species were most likely dealt as *F. prausnitzii* in previous studies. Moreover, a recent report reclassified several strains classified as *F. prausnitzii* as *Faecalibacterium duncaniae* sp. nov. and *Faecalibacterium hattorii* sp. nov. (Sakamoto et al., 2023).

*Faecalibacterium prausnitzii* has been postulated as a next-generation probiotic (Maioli et al., 2021), but its health-promoting properties as non-viable microbe have not yet been published. However, *in vitro* assays demonstrated its postbiotic potential as its extracellular vesicles (membrane and cell wall remains that would fit the definition of postbiotics once a health benefit be demonstrated) promoted the mRNA expression levels of tight junction genes in the human epithelial colorectal adenocarcinoma (Caco-2) cell line (Mosavi et al., 2020). The future will tell if the developers choose to market *Faecalibacterium* as a food ingredient/food supplement or a medicinal product and if they will use the live or inanimate form of the bacteria in the products.

## 3 Regulatory framework of non-viable microbe-based pharmaceutical products in the European Union

Based on the Directive 2004/27/CE, amending the Directive 2001/83/CE, “medicinal products refer to (a) any substance or combination of substances presented as having properties for treating or preventing disease in human beings; or (b) any substance or combination of substances which may be used in or administered to human beings either with a view to restoring, correcting or modifying physiological functions by exerting a pharmacological, immunological or metabolic action, or to making a medical diagnosis”. In this European pharmaceutical regulatory framework, subcategories such as “*biological medicinal products*” are defined. Annex 1, part 1 of the directive 2001/83/CE mentions that “a biological medicinal product is a product, the active substance of which is a biological substance. A biological substance is a substance that is produced by or extracted from a biological source and that needs for its characterisation and the determination of its quality a combination of physico-chemical-biological testing, together with the production process and its control.” Postbiotics, derived from living organisms, can therefore be considered as active substances used in biological medicinal products. So, developers of postbiotics-based medicinal products can rely on all the guidelines dedicated to biological medicinal products even if postbiotics currently have no dedicated guidelines.

Regulatory frameworks and guidelines are based on product(s) currently under evaluation/already evaluated by the competent authorities. Indeed, these authorities are unable to anticipate all the innovations coming from a fast-moving field such as the microbiome field. Likely, regulatory frameworks will evolve as innovative products are developed, with a high priority given to safety of the end-users.

However, regulators should also prioritise establishing frameworks that encourage innovation. When developing innovative products, developers have to be proactive and propose relevant and innovation-specific criteria; regulators can either adopt or reply with further concerns or requests for documentation. Nevertheless, no binding criteria are established before any number of products go through their respective evaluation process. Indeed, the premature adoption of binding criteria may hinder innovation. Developers of innovative products should see this perceived delay in the creation of a dedicated regulatory framework as a signal that they can (and should) act as participants in the development of relevant evaluation criteria and guidelines.

When considering the development of postbiotics, one can anticipate two major challenges. The first is meeting requirements necessary for characterisation of pharmaceutical grade products. Indeed, a postbiotic is “a preparation” and may contain different components (cells, cell fragments and metabolites). Suitable characterisation of the “preparation” is also necessary for pharmacokinetics and pharmacodynamics (PK/PD) studies as well as studies to determine the preparation’s mode of action. The second challenge is related to the potential absorption of these compounds and their systemic distribution by the host. Unlike probiotics developed as medicinal products, known as Live Biotherapeutic Products (LBPs) (Cordaillat-Simmons et al., 2020), where there is very often no systemic absorption and no translocation, in the case of postbiotics, depending on the manufacturing processes and the content of the preparation (bacterial-derived metabolites, bacterial cell wall components, bacterial DNA, etc.), the compounds could be absorbed and associated with immunogenic reactions that will require careful evaluation.

## 4 Examples of products containing postbiotics and regulated as medical devices or medicinal products

The use of inactivated microorganisms in medicinal products is not new. In the dawn of Microbiology in the late 19th Century, the American bone surgeon William B. Coley observed that a mixture of heat-inactivated *Streptococcus pyogenes* and *Serratia marcescens* was efficacious in the treatment of different cancers (McCarthy, 2006). A new, biochemically well-defined, and current good manufacturing practice-compliant preparation has been developed and investigated in patients with NY-ESO-1 expressing cancers (Karbach et al., 2012). Between the 1980s and today, a number of non-viable-microbe-based medicinal products, fitting ISAPP’s definition of postbiotics, have been and continue to be developed and approved in different applications in several European countries. Examples are a product that includes heat-inactivated *Limosilactobacillus fermentum* CNCM MA65/4E-1b plus *Lactobacillus delbrueckii* subsp. *delbrueckii* CNCM MA65/4E-2z and their fermentation metabolites to manage diarrhoea in children (Malagón-Rojas et al., 2020) and a bacterial lysate of *Streptococcus pneumoniae, S. pyogenes, Moraxella catarrhalis, Staphylococcus. aureus, Haeomophilus influenzae* and *Klebsiella pneumoniae* indicated for the prevention of recurrent upper respiratory tract infections in children and adults (Braido et at., 2014).

Although the mechanisms of action by which these postbiotic-based medicinal products function are not fully elucidated, the interaction between these microorganisms or some of their components and the immune system plays a major role (Mazziotta et al., 2023). However, some other postbiotics may have a physical mechanism of action, such as by attaching to intestinal cells (Singh et al., 2017), which may prevent the translocation of pathogens and toxins from the gut lumen to the bloodstream. These postbiotics with a purely physical mechanism of action could, theoretically, be developed as medical devices. Such products are regulated as medical devices. For example, a product based on heat-inactivated *Bifidobacterium bifidum* MIMBb75 to manage irritable bowel syndrome (Andresen et al., 2020) is marketed as a medical device in Europe (CE-marked). Under the EU law (Regulation EU, 2017/745), medical devices are products that prevent, treat or alleviate disease by non-pharmacological, immunological or metabolic means, which are the mechanisms reserved for change medicinal products (Table 1). The use of postbiotics in medical devices in Europe may hold promise, as a new regulatory framework for these products became available in 2021, which stated that medical devices may not contain or consist of […] viable microorganisms, bacteria, fungi or viruses in order to achieve or support the intended purpose of the product (Regulation EU 2017/745), but non-viable microbes are not excluded. However, as stated above, purely physical mechanisms of action are required to comply with the medical device status (Table 1).

## 5 Postbiotics for skin and vaginal applications

Postbiotics are not only explored for gut applications, and indeed have been targeted to other mucosal surfaces of the human host, such as the skin, vagina and nose. For skin applications, postbiotics are often preferred over probiotics, because of the difficulty to formulate live bacteria in skin formulations such as creams and shampoos with sufficient shelf life (Lebeer et al., 2022). Some of the earlier reports on skin postbiotics or lysates originate in Japan (Chiba, 2007), although it is not clear how well these products have been studied in clinical trials. In a recent study in South Korea with the heat-treated postbiotic *Pediococcus acidilactici* LM1013, promising *in vitro* and clinical data were reported for acne vulgaris patients (Bae et al., 2023), although care should be taken with the interpretation, because it was a single-arm clinical trial. In a placebo-controlled recent study in Taiwan, the heat-killed postbiotic *Lacticaseibacillus paracasei* GMNL-653 was able to ameliorate the skin health of the scalp in 22 volunteers when applied in a shampoo (Tsai et al., 2023). An important advantage of postbiotics seems that atypical taxa, that is taxa other than lactic acid bacteria with a long history of safe use, can also be explored as postbiotics for the skin. Several cosmetic companies across the world have postbiotic skin formulations on the market, although the microbial origin and clinical documentation is not always clear from the packaging or website. Clearly, the cosmetic postbiotic industry could benefit from a commitment to more transparency and granularity in labeling as well as more scientific rigor for their postbiotics-based products. Regulation (EC) N° 1223/2009 on cosmetic products is the main regulatory framework for finished cosmetic products when placed on the EU market. This Regulation, entered into force in July 2013, enables further harmonization. Before being placed on the EU market, all cosmetics products must be listed on a centralised database, the Cosmetic Products Notification Portal (CPNP), managed by the European Commission. However, there is no “European Agency,” the equivalent to EFSA or EMA, for cosmetic products. The person or company placing the cosmetic product on the market is responsible for ensuring that this product is safe and complies with the relevant obligations set out in the Regulation (EC) N°1223/2009. This Regulation also makes EU countries responsible for market surveillance at national level.

The situation is different for vaginal and nasal postbiotics. When used in formulations for internal use, they cannot be put on the market as a cosmetic in the EU. Some products containing inanimate microbes appear to be introduced into the market as medical devices. When such postbiotic formulations are used, even temporary colonisation of the vagina with the applied microorganisms is not possible, while this is a key factor for many vaginal probiotics (Oerlemans et al., 2020). However, a recent study showed that a postbiotic gel has benefits, such as increase of endogenous lactobacilli and relief of clinical symptoms of bacterial vaginosis (Shen et al., 2023). Another study in Japan used a postbiotic gel containing *Lacticaseibacillus rhamnosus* vitaP1 and *Lactiplantibacillus plantarum* KCTC3108 in an internal feminine gel product in a randomized controlled pilot study in 35 premenopausal and 35 postmenopausal healthy women (Yoshikata et al., 2022). Researchers also found putatively beneficial changes to the vaginal microbiome, even though live bacteria were not administered. Another study showed the potential of lysates and heat inactivated *Lacticaseibacillus casei* LH23 to suppress human papillomavirus (HPV) infection and its progression into cervical cancer as vaginal postbiotics, but this is only based on *in vitro* work (Hu et al., 2022). These examples show that specific vaginal postbiotics have potential via multiple modes of action, although further documentation of clinical efficacy in high quality trials is needed. It is not always clear from the publications whether the products described are already on the market, and under which regulatory status.

## 6 The marketing opportunity of products based on non-viable microorganisms

Since the ISAPP consensus statement provided a framework for marketers to determine if their products fit the definition, or not, interest in postbiotics in the business-to-business market has boomed and has become a major trend communicated by media focused on biotics. According to Lumina Intelligence (https://www.lumina-intelligence.com/probiotics-reports/postbiotics-new-tools-in-microbiome-modulation/), in the e-commerce of food supplements across 25 countries, postbiotics growth is double-digit (31% growth in 2022 on 2021, and an estimated 21% growth in 2023, in USD terms, fixed exchange rate and constant growth, excluding inflation). United States, South Korea and Japan are driving the growth.

Indeed, postbiotics bring a genuine opportunity to the market in food, beverages, nutraceuticals, pharmaceuticals, cosmetics, and feed. Compared to probiotics, postbiotics may offer: a more favourable safety profile thanks to an absence of risk of infection in vulnerable populations; more complex formulations with other compounds without the risk of these compounds altering the viability of the bacteria (including in food matrices, where, since they do not metabolise nutrients, they do not acidify or modify the taste profile); access to certain product categories which are still challenging regulatory-wise for probiotics in the European Union (such as cosmetics); an easier manipulation in food and pharmaceutical industrial processes, postbiotics often being less sensitive to heat, pressure, blending, grinding (although this is not always the case as the intact cell wall in live bacteria can serve to protect the bacteria); as well as potential extension of the product’s shelf life. One challenge specific to postbiotics is the enumeration throughout product shelf life since the inactivated bacteria cannot grow via culturing methods; however other methods such as flow cytometry already offer solutions.

Postbiotics thus open the door to further applications and opportunities. However, probiotics do not become postbiotics just by inactivation. Their efficacy as inanimate microorganisms must be demonstrated. The opportunity to translate a probiotic benefit to its postbiotic doppelganger depends on the identification of a mechanism of action and the understanding of the microbe’s capacity to deliver the action when dead. Yet, it must be remembered that is not mandatory that the progenitor strain be a probiotic.

Recently, the term ‘metabiotics’ (https://documents.atlantiatrials.com/view/882647320/) was proposed to refer to active metabolites, and such a category could help accelerate the bridge to pharmaceutical applications, as here the microbiome, probiotics, or postbiotics become vectors of active small molecules and speak the language of pharmacology. Yet, we must also be careful to not overpopulate the field with too many terms and definitions. In the ISAPP consensus paper on postbiotics (Salminen et al., 2021a), the panel discussed that metabolites that can be defined by their chemical name do not need a specific term. The market opening also requires attention to translation and education to consumers. FMCG Gurus’ report on prebiotics, probiotics, and postbiotics in 2022 (https://fmcggurus.com/reports/fmcg-gurus-prebiotics-probiotics-postbiotics-global-report-2022/) reported that three-quarters of consumers confound probiotics and prebiotics, and only a small minority have heard of postbiotics.

## 7 Conclusion

The use of non-viable microorganisms, with or without cell components, now defined by ISAPP as postbiotics, is not new in food or pharma but has recently gained a renewed interest and attention by product developers. This is especially the case since approval of postbiotics as novel foods in the European Union. The definition proposed by ISAPP for the term postbiotic is a definition that makes no reference to any particular health benefit, nor any finished product, target population or specific regulatory status, being then flexible enough to accommodate traditional products and innovations in a large range of finished products, from foods and food supplements to medicinal products or medical devices.

## Data Availability

The original contributions presented in the study are included in the article/Supplementary material, further inquiries can be directed to the corresponding author.
